# Automatic Extraction of Nanoparticle Properties Using Natural Language Processing: NanoSifter an Application to Acquire PAMAM Dendrimer Properties

**DOI:** 10.1371/journal.pone.0083932

**Published:** 2014-01-02

**Authors:** David E. Jones, Sean Igo, John Hurdle, Julio C. Facelli

**Affiliations:** 1 Department of Biomedical Informatics, University of Utah, Salt Lake City, Utah, United States of America; 2 Center for High Performance Computing, University of Utah, Salt Lake City, Utah, United States of America; Universidad de Castilla-La Mancha, Spain

## Abstract

In this study, we demonstrate the use of natural language processing methods to extract, from nanomedicine literature, numeric values of biomedical property terms of poly(amidoamine) dendrimers. We have developed a method for extracting these values for properties taken from the NanoParticle Ontology, using the General Architecture for Text Engineering and a Nearly-New Information Extraction System. We also created a method for associating the identified numeric values with their corresponding dendrimer properties, called NanoSifter.

We demonstrate that our system can correctly extract numeric values of dendrimer properties reported in the cancer treatment literature with high recall, precision, and f-measure. The micro-averaged recall was 0.99, precision was 0.84, and f-measure was 0.91. Similarly, the macro-averaged recall was 0.99, precision was 0.87, and f-measure was 0.92. To our knowledge, these results are the first application of text mining to extract and associate dendrimer property terms and their corresponding numeric values.

## Introduction

Nanomedicine is the field of study that considers the application of nanoparticles and nanoscience techniques to health care and medical research [1]. A main focus of nanomedicine includes the use of nanoparticles as delivery vectors for pharmaceutics, diagnostic devices, and tissue replacement materials [2]. This field is relatively new, however it is producing large numbers of publications and substantial new data each year [3]. Data being published contains valuable information regarding how the structure of these nanoparticles relates to their biochemical and biophysical properties, which include but are not limited to their diameter, molecular weight, surface charge, zeta potential, bioavailability, cytotoxicity, etc. [4].

We have chosen dendrimers for our initial application of natural language processing (NLP) to nanomedicine, because they are well-defined, highly branched polymeric nanoparticles that can easily be modified to differing specifications. There is also substantial literature reporting their biological, chemical, and physical properties. Dendrimers are composed of a central core that is surrounded by concentric shells [5], [6]. The number of shells that extend out from the central core determines the particular generation of the dendrimer. Due to their structure, these molecules form very symmetric, three-dimensional particles that promise to be highly useful in the fields of pharmaceutics and medicine as delivery vectors [7]. The scaffold structure of dendrimers has been found to be a suitable carrier for a variety of drugs and siRNA, improving the solubility and bioavailability of poorly soluble agents. Currently there are several classes of dendrimers in use or under consideration for biomedical applications. This study focused on poly(amidoamine) (PAMAM) dendrimers that show promise for cancer treatment.

Databases and repositories containing information relevant to biomedical nanoparticles, especially their biochemical and biophysical properties, are critical for both primary research as well as secondary uses such as data mining and predictive modeling. The American National Standards Institute's Nanotechnology Standards Panel (ANSI-NSP) has created a Nanotechnology Standards database which is a free for individuals and groups seeking information about standards and other relevant documents related to nanomaterials and nanotechnology-related products and processes [8]. The database does not directly host standards and other similar documents, however it provides a place for standards developing organizations to add their relevant documents. This may someday be an important resource for the future development of standardized terminology in the field of nanotechnology and nanomedicine, but it does not contain an extensive collection of values of biological properties of medical nanomaterials.

nanoHUB.org is the premier site for computational nanotechnology research, education, and collaboration [9]. This resource provides an environment for collaboration and aggregation of tools used in simulating nanoscale phenomena. But with this resource, the researchers must provide their own nanomaterial-specific data to utilize the host of simulation tools provided. To our knowledge, there is no authoritative, up-to-date database where researchers consistently contribute results from new publications on biomedical nanoparticles and their properties. Some attempts have been reported in the literature, like caNanoLab, a database created by the National Cancer Institute for sharing nanoparticle information [10]. However, caNanoLab contains a limited number of nanoparticles, and for those it often has incomplete information regarding their biological, chemical, and physical properties. Also, there are only limited capabilities to query this system. No data model exists to support comparing the properties of a molecule to its biochemical and biophysical activity. These properties are necessary to advance research on nanoparticles, but the only way to retrieve this information currently is by manual extraction from the primary literature.

Though manual extraction is a very time consuming and resource intensive process, little research has been done to apply computational methods to obtain nanoparticle property data from the vast biomedical literature on nanoparticles. Information extraction (IE) efforts are widely acknowledged to be important in harnessing the rapid advance of biomedical knowledge, particularly in areas where important factual information is published in diverse literature [11]. In particular, NLP is a family of methods based on syntactic/semantic analysis that can extract information automatically from the literature [12].

NLP has been used effectively in other biomedical domains. For instance, Chaussabel utilized NLP algorithms to extract data from the literature on cell line profiling. He observed that this approach could be applied beyond genomic data analysis [13]. Garten et al. successfully applied NLP methods to the pharmacogenomics literature to create structured databases built on data from unstructured text [14]. Hunter et al. created a system called OpenDMAP that extracts protein transport, interaction, and gene expression assertions [11]. In the field of nanoinformatics there has been an attempt at harnessing the utility of NLP in the nanomedicine literature by Garcia-Remesal and colleagues. They developed a method utilizing named entity recognition to identify four different categories of information: nanoparticle names, routes of exposure, toxic effects, and particle targets [15]. The method that this group developed was moderately successful, but it was designed as a proof-of-concept with limited quantitative detail. Our goal is to gather detailed quantitative data associated with dendrimer properties.

In this study, we evaluate the use of NLP methods to extract numeric values for the properties of biomedical dendrimers reported in the cancer treatment literature. We use open source tools for extracting particle property values, using the NanoParticle Ontology (NPO) [4] as a starting point. In particular, the tools we use are a processing pipeline called the General Architecture for Text Engineering (GATE) and its IE module ANNIE (a Nearly-New Information Extraction System) [16]. In a real-world sentence, a nanoparticle property term can appear arbitrarily far from its associated value, so we also created a method of associating the two. We demonstrate that our system can correctly extract dendrimer property terms and their corresponding numeric values as evaluated by the typical NLP metrics of recall, precision, and f-measure score.

## Materials and Methods

### Literature Corpus

We collected from PubMedCentral relevant articles on dendrimer nanoparticles as reported in the cancer treatment literature. Articles were retrieved in pdf format. The search criteria used was “PAMAM dendrimers AND cancer treatment.” This search yielded 420 journal articles on March 4, 2013. Articles were excluded from this study if they did not contain explicit numeric values of biological, chemical, and/or physical properties of dendrimers. From this pool of 420 articles, we randomly selected 200 journal articles. A subset of 100 articles was used as the training set for our system. The other subset of 100 articles was used for the creation of the test set for our system. Citations for both the training and test set of documents can be found in the supplementary information (Appendix S1 and Appendix S2). For similar applications in related fields, the selection of a test set of approximately 100 documents is a common target that represents a compromise of quality and cost of the manual review. For instance Zaremba et al. used a test set of 138 abstracts to analyze enteropathogenic bacteria, such as *Escherichia coli* and *Salmonella*, literature [17].

### NLP Method Development

The NLP system reported here uses a two-step process to extract the desired property terms and numeric values. The first step involves the actual identification and annotation of the numeric values and dendrimer property terms. This corpus annotation pipeline was built using the Java Annotations Patterns Engine (JAPE) and integrating components from ANNIE within GATE. In order to search for the numeric values, we had to develop a regular expression model (Appendix S3). The specific dendrimer property terms were selected from the NPO and represent the properties of nanoparticles. The dendrimer property terms were selected from the NPO with the ultimate goal of linking the NPO with our tool to provide metadata for the data extractions from the nanomedicine literature. The initial nanoparticle property terms list was confirmed to be relevant for the nanomedicine community by expert review by the members of Dr. Hamidreza S. Ghandehari's research lab (http://nanoinstitute.utah.edu/research/ustar-clusters/ghandehari-lab/ghandehari-PI.php) at the University of Utah. The list of terms considered here includes hydrodynamic diameter (NPO_1915), particle diameter (NPO_1539), molecular weight (NPO_1171), zeta potential (NPO_1302), cytotoxicity (NPO_1340), IC50 (NPO_1195), cell viability (NPO_1343), encapsulation efficiency (NPO_1336), loading efficiency (NPO_1334), and transfection efficiency (NPO_1335). The property terms, their corresponding NPO identification code, and their definitions can be found in Table 1. To search for these property terms, the system utilizes a simple keyword identification scheme.

**Table 1:** Listing of the NPO Property Terms.

PROPERTY TERM	NPO CODE	DEFINITION
Hydrodynamic Diameter	NPO_1915	The hydrodynamic size which is the diameter of a particle or molecule (approximated as a sphere) in an aqueous solution.
Particle Diameter	NPO_1539	Diameter which inheres in a particle.
Molecular Weight	NPO_1171	The sum of the relative atomic masses of the constituent atoms of a molecule.
Zeta Potential	NPO_1302	The potential difference between the bulk dispersion medium (liquid) and the stationary layer of liquid near the surface of the dispersed particulate.
Cytotoxicity	NPO_1340	Toxicity that impairs or damages cells, and it is a desired property of the dispersed particulate.
IC50	NPO_1195	A measure of toxicity which is the concentration of a drug or inhibitor that is required to inhibit a biological process or a participant's activity in that process by half.
Cell Viability	NPO_1343	Viability of a cell to proliferate, grow, divide, or repair damaged cell components.
Encapsulation Efficiency	NPO_1336	The efficiency of inhering in a nanomaterial or supramolecular structure by virtue of its capacity to encapsulate an amount of molecular entity, isotope or nanomaterial.
Loading Efficiency	NPO_1334	A quality inhering in a material entity by virtue of it having the capacity to carry an amount of another material entity.
Transfection Efficiency	NPO_1335	The efficiency inhering in a bearer's ability to facilitate transfection.

The training set of documents was manually annotated for numeric values and dendrimer property terms using GATE. Following the annotation, the numeric values associated with each property term were extracted manually and organized in a tabular format for ease of use and comparison. Once the pipeline was able to successfully annotate the numeric values and the dendrimer property terms, we developed an algorithm that would associate numeric values and dendrimer property terms that occurred within the same sentence using proximity metrics. We selected a proximity distance metric of 200 characters because our preliminary experiments have shown that the sensitivity and specificity of the system was best for this distance in the training set. For instance we observed that if we increased it, the number of false positives increased without any improvement in the observed recall of the system. Finally, we optimized performance iteratively before moving on to the test set of documents.

### Reference Standard Creation

Two domain experts were selected from the nanotechnology program at the University of Utah. Before allowing them to review the test subset of 100 articles, they independently reviewed, annotated, and extracted information from the training set of articles using GATE. The annotations consisted of numeric values and dendrimer property terms selected from the NPO. Their annotations were compared and Cohen's kappa was calculated. Cohen's kappa is a statistical measure of inter-rater reliability, and for this study we required it to be ≥80%, which has been categorized as excellent by Fleiss at a value of 75% or higher [18].

Upon achieving an inter-rater reliability of 80%, the annotators independently reviewed, annotated, and extracted information from the test set of articles. Again, the numeric values and dendrimer property terms were taken from the NPO and were annotated using GATE. Following the annotation, the numeric values associated with each property term were extracted and organized in a tabular format.

### NLP System Performance

The subset of 100 test articles was processed by our new NLP system. The output from the system was organized in a tabular format for ease of use and comparison.

### Data Analysis

Our NLP and manual results were compared on a by-nanoparticle property term basis. The extracted numeric values associated to the dendrimer property terms were evaluated and determined to be true positive, false positive, or false negative. First, we calculated the recall, precision, and f-measure of each nanoparticle property term. We then calculated the micro-averaged and macro-averaged recall, precision, and f-measure. When using micro-averaged measurements, each “source” (e.g. document) is given the same weight, and calculations are made on a pooled contingency table [19]. Macro-averaged measurements are calculated by giving the same weight to each concept category or class (e.g., dendrimer property term) [19].

The recall, precision, and f-measure were calculated using the following equations: 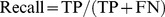
(1)

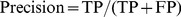
(2)

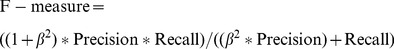
(3)


In these equations TP is true positive, FP is false positive, FN is false negative, and β is the weighting applied to the relationship between precision and recall. For our purposes we decided to weight the precision and recall evenly, so β = 1.

## Results

Table 2 summarizes the results of the evaluation of the NLP system that we created. The results of the system are compared against the manually annotated reference standard. The table shows the recall, precision, and f-measure for each of the nanoparticle property terms and numeric value relationships. Table 3 displays both the micro-averaged and macro-averaged recall, precision, and f-measure values.

**Table 2:** Results from the Evaluation of the Nanosifter NLP System.

Nanoparticle Property Term	TP	FP	FN	Recall	Precision	F-measure	Occurrences by Article
Hydrodynamic Diameter	8	0	0	1	1	1	6
Particle Diameter	211	39	1	0.995283	0.844	0.91341991	56
Molecular Weight	143	23	2	0.986207	0.86145	0.91961415	25
Zeta Potential	41	0	1	0.97619	1	0.98795181	16
Cytotoxicity	124	18	1	0.992	0.87324	0.92883895	29
IC50	47	8	1	0.979167	0.85455	0.91262136	15
Cell Viability	78	31	0	1	0.7156	0.8342246	25
Encapsulation Efficiency	1	0	0	1	1	1	1
Loading Efficiency	5	0	0	1	1	1	1
Transfection Efficiency	19	13	1	0.95	0.59375	0.73076923	9

**Table 3:** Micro-averaged and Macro-averaged Recall, Precision, and F-measure

Type of Average	Recall	Precision	F-measure
Micro	0.989766	0.83684	0.90689886
Macro	0.987885	0.87426	0.922744

As can be seen in Table 2, our NLP system yields recall values ranging from 0.95 to 1 and precision values ranging from 0.59 to 1. The f-measure values range from 0.73 to 1. The micro-averaged values for recall was 0.99, precision was 0.84, and f-measure was 0.91. Similarly, the macro-averaged values for recall was 0.99, precision was 0.87, and f-measure was 0.92.

## Discussion

The tables show an important difference between recall and precision. In this task, high recall is preferred to high precision, because we do not want our system to miss instances of property terms and their associated numeric values. The number of articles returned for any given search (e.g., our “PAMAM dendrimers AND cancer treatment” search) is too large for routine manual search, but reviewing NanoSifter *results* is quite traceable. The results can be manually reviewed post-processing without much additional effort. From the results, it can be seen that “encapsulation efficiency” and “loading efficiency” were the best property terms extracted with recall, precision, and f-measure values of 1. These scores are likely due to the low prevalence of these properties appearing in our literature corpus. “Transfection efficiency” was the property term that was the least well extracted from nanomedicine literature. It had a recall value of 0.95, a precision value of 0.59, and an f-measure value of 0.73.

These results indicate that the NanoSifter NLP system can, generally, extract numeric values associated with particle property terms from dendrimers reported in the cancer treatment literature with high recall, precision, and f-measure scores. To the authors' knowledge, these results are the first application of text mining to extract numeric values associated to dendrimer property terms from nanomedicine literature. With regards to our application, the high recall values are more important than the moderate precision values. This is because the lack of precision is manageable and can be quickly corrected by manual post processing of the annotated text.

As can be seen from the results, there was a fair amount of fluctuation in the values for precision for each property term. There were a few property terms that yielded precisions of 1 including “hydrodynamic diameter,” “zeta potential,” “encapsulation efficiency,” and “loading efficiency.” This can be accounted for by the limited number of instances that these terms appeared in the literature. Of all of the property terms used in this study, these were the least common. The next tier of precision values of interest are those that were greater than 0.80, these include “particle diameter,” “molecular weight,” “cytotoxicity,” and “IC50.” These property terms yielded quite reasonable precision values, as we expected based upon their occurrences in the literature and the specificity of the syntax used when describing these property terms and their numeric values.

The lowest precision values could be seen for “cell viability” (0.72) and “transfection efficiency” (0.59). One reason for these lower precision values is that the numeric units for these properties are percentages. There was a significant number of false positives in the literature corpus because the number of occurrences of percentages for other, non-particle items within the 200-character proximity metric was large. With specific regard to “transfection efficiency,” precision values for this term were the lowest because the terminology used to refer to this property is not standardized. There are many different ways in which the literature refers to this property, making it difficult not to overfit a method of retrieving the numeric values of this property.

### Limitations

NanoSifter uses a method that appears to be generally reliable and accurate. However, there are imperfections that were observed while processing and analyzing the data from this study. First, the data extracted by our method is not always directly associated with a dendrimer nanoparticle. For instance, many times the system correctly finds, annotates, and extracts a “molecular weight measurement”, but this measurement may be associated with a subunit utilized in the synthesis of a PAMAM dendrimer or another material used in one of the articles. A method to address this limitation could include post-analysis manual review of the system's performance. Another limitation of our system is that the NanoSifter algorithm can only pair a nanoparticle property term with a single numeric value annotation before and after itself. This causes a problem when a sentence is more complex and contains a property term, random text, numeric value, random text, or another numeric value. In NLP, this is a problem called co-reference resolution, and it could be addressed with a more sophisticated language model than the one used in this study.

Another limitation is that our system would only retrieve the first numeric value expressed following the property term. This situation accounts for some of the false negatives (“particle diameter,” “cytotoxicity,” “IC50,” and “transfection efficiency”) found in our analysis. This could also be addressed by using a more sophisticated language model than the one used in this study. Finally, the other false negatives, “molecular weight” and “zeta potential,” account for another limitation of our system. Since we were processing pdf documents in this study, occasionally there would be an instance where a property term exceeded a single line of text, so a dash would be inserted in the word and it would continue on the next line. The method used in developing this system did not account for this artifact. Therefore the NanoSifter NLP system would not annotate this property term, and no association would be made to the corresponding numeric value. A method for addressing this would be to use XML documents instead of pdfs in future analyses. These limitations are not novel to our approach, as they are common throughout the field of NLP. Nonetheless they are counterbalanced by the ability to extract information from journal articles at a much lower cost than manual review.

### Future Work

Since this is early work in an important but neglected area of nanoinformatics, there are many directions this research could be taken. The first priority will be to make corrections to our system to try to improve our recall, precision, and f-measure values. Another priority will be to attempt to use this system to annotate and extract information from another subclass of nanoparticles. This will help to validate the ability of this system to generalize across the field of nanoparticles. One of the most important next steps would be to expand the property terms and numeric values that the system targets. Some specific properties that we are considering include “exposure times” and “cell types” interacting with the nanoparticles. This would allow for greater databases to be created regarding PAMAM dendrimers and nanoparticles in general. Another goal would be to more seamlessly integrate the NPO into our system so that the annotations and extractions contain descriptive metadata. Finally, it is important that we attempt to implement some sort of negation analysis tool into our system. This would specifically help in the instances where an article states that the dendrimer nanoparticles were not toxic at a certain concentration.

## Conclusion

In this paper, we have presented a nanoinformatics method based on NLP approaches for automatically extracting numeric values associated with dendrimer property terms from the nanomedicine literature. The results from our analysis demonstrate that the NanoSifter NLP system can be used to reliably and accurately extract information from dendrimers developed for cancer treatment literature and shows promise for the future of text mining in the field of nanoinformatics. This initial research in the field of applying NLP to nanomedicine literature could assist in significant advances for the nanomedicine community. This work could lead to the creation of databases containing valuable information regarding nanoparticles at a much lower cost than using manual review. The readily available data on nanomedical relevant particles could be further analyzed for many secondary uses of the data. In particular, the acquired data could be used for data mining to find correlations between properties, create predictive models like quantitative structure activity relationships, and eventually reach the point where potential candidate molecules can be created *in silico* and modeled to theoretically predict their biochemical activity before synthesis. This would reduce the search space for novel, effective nanoparticles for use in medicine and pharmaceutics.

## Supporting Information

Appendix S1 **Document containing the citations for the training set corpus.**
(DOCX)

Appendix S2 **Document containing the citations for the test set corpus.**
(DOCX)

Appendix S3 **Regular expression model JAPE file for identifying numeric values in the literature.**
(JAPE)
