# Supplementary material for: Automatic Extraction of Nanoparticle Properties Using Natural Language Processing: NanoSifter an Application to Acquire PAMAM Dendrimer Properties
Source: PLoS One. 2014 Jan 2;9(1):e83932. doi: 10.1371/journal.pone.0083932 (PMC3879259; doi:10.1371/journal.pone.0083932)
Supplement: Appendix S1 — Document containing the citations for the training set corpus. (DOCX) [file pone.0083932.s001.docx]

**Training Set Documents**

[[1-100](#_ENREF_1)]

1. Agrawal A, Min DH, Singh N, Zhu H, Birjiniuk A, et al. (2009) Functional delivery of siRNA in mice using dendriworms. ACS Nano 3: 2495-2504.

2. Aillon KL, Xie Y, El-Gendy N, Berkland CJ, Forrest ML (2009) Effects of nanomaterial physicochemical properties on in vivo toxicity. Adv Drug Deliv Rev 61: 457-466.

3. Alcala MA, Kwan SY, Shade CM, Lang M, Uh H, et al. (2011) Luminescence targeting and imaging using a nanoscale generation 3 dendrimer in an in vivo colorectal metastatic rat model. Nanomedicine (Lond) 7: 249-258.

4. Alcala MA, Shade CM, Uh H, Kwan SY, Bischof M, et al. (2011) Preferential accumulation within tumors and in vivo imaging by functionalized luminescent dendrimer lanthanide complexes. Biomaterials 32: 9343-9352.

5. Ali MM, Woods M, Caravan P, Opina AC, Spiller M, et al. (2008) Synthesis and relaxometric studies of a dendrimer-based pH-responsive MRI contrast agent. Chemistry 14: 7250-7258.

6. Al-Jamal KT, Al-Jamal WT, Akerman S, Podesta JE, Yilmazer A, et al. (2010) Systemic antiangiogenic activity of cationic poly-L-lysine dendrimer delays tumor growth. Proc Natl Acad Sci U S A 107: 3966-3971.

7. Ambade AV, Savariar EN, Thayumanavan S (2005) Dendrimeric micelles for controlled drug release and targeted delivery. Mol Pharm 2: 264-272.

8. Arima H, Motoyama K (2009) Recent Findings Concerning PAMAM Dendrimer Conjugates with Cyclodextrins as Carriers of DNA and RNA. Sensors (Basel) 9: 6346-6361.

9. Biswal BK, Kavitha M, Verma RS, Prasad E (2009) Tumor cell imaging using the intrinsic emission from PAMAM dendrimer: a case study with HeLa cells. Cytotechnology 61: 17-24.

10. Bonner DK, Leung C, Chen-Liang J, Chingozha L, Langer R, et al. (2011) Intracellular trafficking of polyamidoamine-poly(ethylene glycol) block copolymers in DNA delivery. Bioconjug Chem 22: 1519-1525.

11. Borgman MP, Aras O, Geyser-Stoops S, Sausville EA, Ghandehari H (2009) Biodistribution of HPMA copolymer-aminohexylgeldanamycin-RGDfK conjugates for prostate cancer drug delivery. Mol Pharm 6: 1836-1847.

12. Chao X, Zhang Z, Guo L, Zhu J, Peng M, et al. (2012) A novel magnetic nanoparticle drug carrier for enhanced cancer chemotherapy. PLoS One 7: e40388.

13. Cheng Z, Thorek DL, Tsourkas A (2010) Gadolinium-conjugated dendrimer nanoclusters as a tumor-targeted T1 magnetic resonance imaging contrast agent. Angew Chem Int Ed Engl 49: 346-350.

14. Choi SK, Verma M, Silpe J, Moody RE, Tang K, et al. (2012) A photochemical approach for controlled drug release in targeted drug delivery. Bioorg Med Chem 20: 1281-1290.

15. Daniels TR, Bernabeu E, Rodriguez JA, Patel S, Kozman M, et al. (2012) The transferrin receptor and the targeted delivery of therapeutic agents against cancer. Biochim Biophys Acta 1820: 291-317.

16. Duan S, Cai S, Yang Q, Forrest ML (2012) Controlled release of nitric oxide chemotherapy using a nano-sized biodegradable multi-arm polymer. Biomaterials 33: 3243-3253.

17. Esipova TV, Ye X, Collins JE, Sakadzic S, Mandeville ET, et al. (2012) Dendritic upconverting nanoparticles enable in vivo multiphoton microscopy with low-power continuous wave sources. Proc Natl Acad Sci U S A 109: 20826-20831.

18. Fox ME, Guillaudeu S, Frechet JM, Jerger K, Macaraeg N, et al. (2009) Synthesis and in vivo antitumor efficacy of PEGylated poly(l-lysine) dendrimer-camptothecin conjugates. Mol Pharm 6: 1562-1572.

19. Garner AL, Park J, Zakhari JS, Lowery CA, Struss AK, et al. (2011) A multivalent probe for AI-2 quorum-sensing receptors. J Am Chem Soc 133: 15934-15937.

20. Gu SZ, Zhao XH, Zhang LX, Li L, Wang ZY, et al. (2009) Anti-angiogenesis effect of generation 4 polyamidoamine/vascular endothelial growth factor antisense oligodeoxynucleotide on breast cancer in vitro. J Zhejiang Univ Sci B 10: 159-167.

21. Hamidi A, Sharifi S, Davaran S, Ghasemi S, Omidi Y, et al. (2012) Novel aldehyde-terminated dendrimers; synthesis and cytotoxicity assay. Bioimpacts 2: 97-103.

22. Holden CA, Yuan Q, Yeudall WA, Lebman DA, Yang H (2010) Surface engineering of macrophages with nanoparticles to generate a cell-nanoparticle hybrid vehicle for hypoxia-targeted drug delivery. Int J Nanomedicine 5: 25-36.

23. Hom C, Lu J, Tamanoi F (2009) Silica nanoparticles as a delivery system for nucleic acid-based reagents. J Mater Chem 19: 6308-6316.

24. Hong L, Krishnamachari Y, Seabold D, Joshi V, Schneider G, et al. (2011) Intracellular release of 17-beta estradiol from cationic polyamidoamine dendrimer surface-modified poly (lactic-co-glycolic acid) microparticles improves osteogenic differentiation of human mesenchymal stromal cells. Tissue Eng Part C Methods 17: 319-325.

25. Jacobson KA (2010) GPCR ligand-dendrimer (GLiDe) conjugates: future smart drugs? Trends Pharmacol Sci 31: 575-579.

26. Jain S, Pitoc GA, Holl EK, Zhang Y, Borst L, et al. (2012) Nucleic acid scavengers inhibit thrombosis without increasing bleeding. Proc Natl Acad Sci U S A 109: 12938-12943.

27. Kasman LM, Barua S, Lu P, Rege K, Voelkel-Johnson C (2009) Polymer-enhanced adenoviral transduction of CAR-negative bladder cancer cells. Mol Pharm 6: 1612-1619.

28. Kelly CV, Leroueil PR, Nett EK, Wereszczynski JM, Baker JR, Jr., et al. (2008) Poly(amidoamine) dendrimers on lipid bilayers I: Free energy and conformation of binding. J Phys Chem B 112: 9337-9345.

29. Kelly CV, Liroff MG, Triplett LD, Leroueil PR, Mullen DG, et al. (2009) Stoichiometry and Structure of Poly(amidoamine) Dendrimer-Lipid Complexes. ACS Nano 3: 1886-1896.

30. Kim Y, Klutz AM, Hechler B, Gao ZG, Gachet C, et al. (2009) Application of the functionalized congener approach to dendrimer-based signaling agents acting through A(2A) adenosine receptors. Purinergic Signal 5: 39-50.

31. Klutz AM, Gao ZG, Lloyd J, Shainberg A, Jacobson KA (2008) Enhanced A3 adenosine receptor selectivity of multivalent nucleoside-dendrimer conjugates. J Nanobiotechnology 6: 12.

32. Klutz K, Schaffert D, Willhauck MJ, Grunwald GK, Haase R, et al. (2011) Epidermal growth factor receptor-targeted (131)I-therapy of liver cancer following systemic delivery of the sodium iodide symporter gene. Mol Ther 19: 676-685.

33. Kobayashi H, Ogawa M, Kosaka N, Choyke PL, Urano Y (2009) Multicolor imaging of lymphatic function with two nanomaterials: quantum dot-labeled cancer cells and dendrimer-based optical agents. Nanomedicine (Lond) 4: 411-419.

34. Krishna AD, Mandraju RK, Kishore G, Kondapi AK (2009) An efficient targeted drug delivery through apotransferrin loaded nanoparticles. PLoS One 4: e7240.

35. Kumar TS, Mishra S, Deflorian F, Yoo LS, Phan K, et al. (2011) Molecular probes for the A2A adenosine receptor based on a pyrazolo[4,3-e][1,2,4]triazolo[1,5-c]pyrimidin-5-amine scaffold. Bioorg Med Chem Lett 21: 2740-2745.

36. Kurtoglu YE, Navath RS, Wang B, Kannan S, Romero R, et al. (2009) Poly(amidoamine) dendrimer-drug conjugates with disulfide linkages for intracellular drug delivery. Biomaterials 30: 2112-2121.

37. Lee CY, Sharma A, Uzarski RL, Cheong JE, Xu H, et al. (2011) Potent antioxidant dendrimers lacking pro-oxidant activity. Free Radic Biol Med 50: 918-925.

38. Lee H, Baker JR, Jr., Larson RG (2006) Molecular dynamics studies of the size, shape, and internal structure of 0% and 90% acetylated fifth-generation polyamidoamine dendrimers in water and methanol. J Phys Chem B 110: 4014-4019.

39. Lee H, Larson RG (2009) Molecular dynamics study of the structure and interparticle interactions of polyethylene glycol-conjugated PAMAM dendrimers. J Phys Chem B 113: 13202-13207.

40. Lee H, Larson RG (2009) Multiscale modeling of dendrimers and their interactions with bilayers and polyelectrolytes. Molecules 14: 423-438.

41. Lee I, Majoros IJ, Williams CR, Athey BD, Baker JR (2009) Interactive Design Strategy for a Multi-Functional PAMAM Dendrimer-Based Nano-Therapeutic Using Computational Models and Experimental Analysis. J Comput Theor Nanosci 6: 54-60.

42. Lee J, Sohn JW, Zhang Y, Leong KW, Pisetsky D, et al. (2011) Nucleic acid-binding polymers as anti-inflammatory agents. Proc Natl Acad Sci U S A 108: 14055-14060.

43. Lesniak WG, Kariapper MS, Nair BM, Tan W, Hutson A, et al. (2007) Synthesis and characterization of PAMAM dendrimer-based multifunctional nanodevices for targeting alphavbeta3 integrins. Bioconjug Chem 18: 1148-1154.

44. Li L, Orner BP, Huang T, Hinck AP, Kiessling LL (2010) Peptide ligands that use a novel binding site to target both TGF-beta receptors. Mol Biosyst 6: 2392-2402.

45. Lo ST, Stern S, Clogston JD, Zheng J, Adiseshaiah PP, et al. (2010) Biological assessment of triazine dendrimer: toxicological profiles, solution behavior, biodistribution, drug release and efficacy in a PEGylated, paclitaxel construct. Mol Pharm 7: 993-1006.

46. Lu Y, Sun B, Li C, Schoenfisch MH (2011) Structurally Diverse Nitric Oxide-Releasing Poly(propylene Imine) Dendrimers. Chem Mater 23: 4227-4233.

47. Luo H, Jiang B, Li B, Li Z, Jiang BH, et al. (2012) Kaempferol nanoparticles achieve strong and selective inhibition of ovarian cancer cell viability. Int J Nanomedicine 7: 3951-3959.

48. Majoros IJ, Williams CR, Becker A, Baker JR, Jr. (2009) Methotrexate delivery via folate targeted dendrimer-based nanotherapeutic platform. Wiley Interdiscip Rev Nanomed Nanobiotechnol 1: 502-510.

49. Majoros IJ, Williams CR, Tomalia DA, Baker JR, Jr. (2008) New Dendrimers: Synthesis and Characterization of Popam - Pamam Hybrid Dendrimers. Macromolecules 41: 8372-8379.

50. McNerny DQ, Kukowska-Latallo JF, Mullen DG, Wallace JM, Desai AM, et al. (2009) RGD dendron bodies; synthetic avidity agents with defined and potentially interchangeable effector sites that can substitute for antibodies. Bioconjug Chem 20: 1853-1859.

51. Mecke A, Lee DK, Ramamoorthy A, Orr BG, Holl MM (2005) Synthetic and natural polycationic polymer nanoparticles interact selectively with fluid-phase domains of DMPC lipid bilayers. Langmuir 21: 8588-8590.

52. Merkel OM, Zheng M, Mintzer MA, Pavan GM, Librizzi D, et al. (2011) Molecular modeling and in vivo imaging can identify successful flexible triazine dendrimer-based siRNA delivery systems. J Control Release 153: 23-33.

53. Mullen DG, Borgmeier EL, Desai AM, van Dongen MA, Barash M, et al. (2010) Isolation and characterization of dendrimers with precise numbers of functional groups. Chemistry 16: 10675-10678.

54. Myung JH, Gajjar KA, Saric J, Eddington DT, Hong S (2011) Dendrimer-mediated multivalent binding for the enhanced capture of tumor cells. Angew Chem Int Ed Engl 50: 11769-11772.

55. Navath RS, Wang B, Kannan S, Romero R, Kannan RM (2010) Stimuli-responsive star poly(ethylene glycol) drug conjugates for improved intracellular delivery of the drug in neuroinflammation. J Control Release 142: 447-456.

56. Nwe K, Bernardo M, Regino CA, Williams M, Brechbiel MW (2010) Comparison of MRI properties between derivatized DTPA and DOTA gadolinium-dendrimer conjugates. Bioorg Med Chem 18: 5925-5931.

57. Nwe K, Bryant LH, Jr., Brechbiel MW (2010) Poly(amidoamine) dendrimer based MRI contrast agents exhibiting enhanced relaxivities derived via metal preligation techniques. Bioconjug Chem 21: 1014-1017.

58. Nwe K, Milenic D, Bryant LH, Regino CA, Brechbiel MW (2011) Preparation, characterization and in vivo assessment of Gd-albumin and Gd-dendrimer conjugates as intravascular contrast-enhancing agents for MRI. J Inorg Biochem 105: 722-727.

59. Nwe K, Xu H, Regino CA, Bernardo M, Ileva L, et al. (2009) A new approach in the preparation of dendrimer-based bifunctional diethylenetriaminepentaacetic acid MR contrast agent derivatives. Bioconjug Chem 20: 1412-1418.

60. Ogawa M, Regino CA, Marcelino B, Williams M, Kosaka N, et al. (2010) New nanosized biocompatible MR contrast agents based on lysine-dendri-graft macromolecules. Bioconjug Chem 21: 955-960.

61. Patel DA, Henry JE, Good TA (2007) Attenuation of beta-amyloid-induced toxicity by sialic-acid-conjugated dendrimers: role of sialic acid attachment. Brain Res 1161: 95-105.

62. Perez AP, Mundina-Weilenmann C, Romero EL, Morilla MJ (2012) Increased brain radioactivity by intranasal P-labeled siRNA dendriplexes within in situ-forming mucoadhesive gels. Int J Nanomedicine 7: 1373-1385.

63. Portevin D, Poupot M, Rolland O, Turrin CO, Fournie JJ, et al. (2009) Regulatory activity of azabisphosphonate-capped dendrimers on human CD4+ T cell proliferation enhances ex-vivo expansion of NK cells from PBMCs for immunotherapy. J Transl Med 7: 82.

64. Qi R, Mullen DG, Baker JR, Holl MM (2010) The mechanism of polyplex internalization into cells: testing the GM1/caveolin-1 lipid raft mediated endocytosis pathway. Mol Pharm 7: 267-279.

65. Qiu B, Ji M, Song X, Zhu Y, Wang Z, et al. (2012) Co-delivery of docetaxel and endostatin by a biodegradable nanoparticle for the synergistic treatment of cervical cancer. Nanoscale Res Lett 7: 666.

66. Regino CA, Walbridge S, Bernardo M, Wong KJ, Johnson D, et al. (2008) A dual CT-MR dendrimer contrast agent as a surrogate marker for convection-enhanced delivery of intracerebral macromolecular therapeutic agents. Contrast Media Mol Imaging 3: 2-8.

67. Ruan J, Shen J, Wang Z, Ji J, Song H, et al. (2011) Efficient preparation and labeling of human induced pluripotent stem cells by nanotechnology. Int J Nanomedicine 6: 425-435.

68. Samuelson LE, Dukes MJ, Hunt CR, Casey JD, Bornhop DJ (2009) TSPO targeted dendrimer imaging agent: synthesis, characterization, and cellular internalization. Bioconjug Chem 20: 2082-2089.

69. Santhakumaran LM, Thomas T, Thomas TJ (2004) Enhanced cellular uptake of a triplex-forming oligonucleotide by nanoparticle formation in the presence of polypropylenimine dendrimers. Nucleic Acids Res 32: 2102-2112.

70. Scherer RL, VanSaun MN, McIntyre JO, Matrisian LM (2008) Optical imaging of matrix metalloproteinase-7 activity in vivo using a proteolytic nanobeacon. Mol Imaging 7: 118-131.

71. Schlick KH, Morgan JR, Weiel JJ, Kelsey MS, Cloninger MJ (2011) Clusters of ligands on dendrimer surfaces. Bioorg Med Chem Lett 21: 5078-5083.

72. Sena LM, Fishman SJ, Jenkins KJ, Xu H, Brechbiel MW, et al. (2010) Magnetic resonance lymphangiography with a nano-sized gadolinium-labeled dendrimer in small and large animal models. Nanomedicine (Lond) 5: 1183-1191.

73. Shi L, Fleming CJ, Riechers SL, Yin NN, Luo J, et al. (2011) High-Resolution Imaging of Dendrimers Used in Drug Delivery via Scanning Probe Microscopy. J Drug Deliv 2011: 254095.

74. Shi X, Lee I, Chen X, Shen M, Xiao S, et al. (2010) Influence of dendrimer surface charge on the bioactivity of 2-methoxyestradiol complexed with dendrimers. Soft Matter 6: 2539-2545.

75. Shi X, Wang SH, Van Antwerp ME, Chen X, Baker JR, Jr. (2009) Targeting and detecting cancer cells using spontaneously formed multifunctional dendrimer-stabilized gold nanoparticles. Analyst 134: 1373-1379.

76. Shukla R, Thomas TP, Peters JL, Desai AM, Kukowska-Latallo J, et al. (2006) HER2 specific tumor targeting with dendrimer conjugated anti-HER2 mAb. Bioconjug Chem 17: 1109-1115.

77. Stasko NA, Fischer TH, Schoenfisch MH (2008) S-nitrosothiol-modified dendrimers as nitric oxide delivery vehicles. Biomacromolecules 9: 834-841.

78. Sun C, Tang T, Uludag H, Cuervo JE (2011) Molecular dynamics simulations of DNA/PEI complexes: effect of PEI branching and protonation state. Biophys J 100: 2754-2763.

79. Swanson SD, Kukowska-Latallo JF, Patri AK, Chen C, Ge S, et al. (2008) Targeted gadolinium-loaded dendrimer nanoparticles for tumor-specific magnetic resonance contrast enhancement. Int J Nanomedicine 3: 201-210.

80. Tarallo R, Carberry TP, Falanga A, Vitiello M, Galdiero S, et al. (2013) Dendrimers functionalized with membrane-interacting peptides for viral inhibition. Int J Nanomedicine 8: 521-534.

81. Taratula O, Garbuzenko OB, Kirkpatrick P, Pandya I, Savla R, et al. (2009) Surface-engineered targeted PPI dendrimer for efficient intracellular and intratumoral siRNA delivery. J Control Release 140: 284-293.

82. Thomas TP, Goonewardena SN, Majoros IJ, Kotlyar A, Cao Z, et al. (2011) Folate-targeted nanoparticles show efficacy in the treatment of inflammatory arthritis. Arthritis Rheum 63: 2671-2680.

83. Thomas TP, Shukla R, Kotlyar A, Kukowska-Latallo J, Baker JR, Jr. (2010) Dendrimer-based tumor cell targeting of fibroblast growth factor-1. Bioorg Med Chem Lett 20: 700-703.

84. Trogden BG, Kim SH, Lee S, Katzenellenbogen JA (2009) Tethered indoles as functionalizable ligands for the estrogen receptor. Bioorg Med Chem Lett 19: 485-488.

85. Tyssen D, Henderson SA, Johnson A, Sterjovski J, Moore K, et al. (2010) Structure activity relationship of dendrimer microbicides with dual action antiviral activity. PLoS One 5: e12309.

86. van der Poll DG, Kieler-Ferguson HM, Floyd WC, Guillaudeu SJ, Jerger K, et al. (2010) Design, synthesis, and biological evaluation of a robust, biodegradable dendrimer. Bioconjug Chem 21: 764-773.

87. Wang AZ, Gu F, Zhang L, Chan JM, Radovic-Moreno A, et al. (2008) Biofunctionalized targeted nanoparticles for therapeutic applications. Expert Opin Biol Ther 8: 1063-1070.

88. Wang H, Shi HB, Yin SK (2011) Polyamidoamine dendrimers as gene delivery carriers in the inner ear: How to improve transfection efficiency. Exp Ther Med 2: 777-781.

89. Wang H, Zheng L, Guo R, Peng C, Shen M, et al. (2012) Dendrimer-entrapped gold nanoparticles as potential CT contrast agents for blood pool imaging. Nanoscale Res Lett 7: 190.

90. Witte AB, Timmer CM, Gam JJ, Choi SK, Banaszak Holl MM, et al. (2012) Biophysical characterization of a riboflavin-conjugated dendrimer platform for targeted drug delivery. Biomacromolecules 13: 507-516.

91. Wu XH, Lu Y, Fang YW, Jiang YX (2012) The polyamidoamine-mediated inhibition of bcl-2 by small hairpin RNA to induce apoptosis in human lens epithelial cells. Mol Vis 18: 74-80.

92. Yallapu MM, Ebeling MC, Chauhan N, Jaggi M, Chauhan SC (2011) Interaction of curcumin nanoformulations with human plasma proteins and erythrocytes. Int J Nanomedicine 6: 2779-2790.

93. Yang H (2010) Nanoparticle-mediated brain-specific drug delivery, imaging, and diagnosis. Pharm Res 27: 1759-1771.

94. Yang H, Kao WJ (2007) Synthesis and characterization of nanoscale dendritic RGD clusters for potential applications in tissue engineering and drug delivery. Int J Nanomedicine 2: 89-99.

95. Yin P, Wang Y, Qiu Y, Hou L, Liu X, et al. (2012) Bufalin-loaded mPEG-PLGA-PLL-cRGD nanoparticles: preparation, cellular uptake, tissue distribution, and anticancer activity. Int J Nanomedicine 7: 3961-3969.

96. Yousefpour P, Atyabi F, Vasheghani-Farahani E, Movahedi AA, Dinarvand R (2011) Targeted delivery of doxorubicin-utilizing chitosan nanoparticles surface-functionalized with anti-Her2 trastuzumab. Int J Nanomedicine 6: 1977-1990.

97. Yuan Q, Fu Y, Kao WJ, Janigro D, Yang H (2011) Transbuccal Delivery of CNS Therapeutic Nanoparticles: Synthesis, Characterization, and In Vitro Permeation Studies. ACS Chem Neurosci 2: 676-683.

98. Zhong YJ, Shao LH, Li Y (2013) Cathepsin B-cleavable doxorubicin prodrugs for targeted cancer therapy (Review). Int J Oncol 42: 373-383.

99. Zhou X, Turchi C, Wang D (2009) Carbohydrate cluster microarrays fabricated on three-dimensional dendrimeric platforms for functional glycomics exploration. J Proteome Res 8: 5031-5040.

100. Zhu W, Okollie B, Bhujwalla ZM, Artemov D (2008) PAMAM dendrimer-based contrast agents for MR imaging of Her-2/neu receptors by a three-step pretargeting approach. Magn Reson Med 59: 679-685.
