# Supplementary material for: Automatic Extraction of Nanoparticle Properties Using Natural Language Processing: NanoSifter an Application to Acquire PAMAM Dendrimer Properties
Source: PLoS One. 2014 Jan 2;9(1):e83932. doi: 10.1371/journal.pone.0083932 (PMC3879259; doi:10.1371/journal.pone.0083932)
Supplement: Appendix S2 — Document containing the citations for the test set corpus. (DOCX) [file pone.0083932.s002.docx]

**Test Set Documents**

[[1-100](#_ENREF_1)]

1. Abderrezak A, Bourassa P, Mandeville JS, Sedaghat-Herati R, Tajmir-Riahi HA (2012) Dendrimers bind antioxidant polyphenols and cisplatin drug. PLoS One 7: e33102.

2. Avila-Salas F, Sandoval C, Caballero J, Guinez-Molinos S, Santos LS, et al. (2012) Study of interaction energies between the PAMAM dendrimer and nonsteroidal anti-inflammatory drug using a distributed computational strategy and experimental analysis by ESI-MS/MS. J Phys Chem B 116: 2031-2039.

3. Barata TS, Teo I, Brocchini S, Zloh M, Shaunak S (2011) Partially glycosylated dendrimers block MD-2 and prevent TLR4-MD-2-LPS complex mediated cytokine responses. PLoS Comput Biol 7: e1002095.

4. Barrett T, Ravizzini G, Choyke PL, Kobayashi H (2009) Dendrimers in medical nanotechnology. IEEE Eng Med Biol Mag 28: 12-22.

5. Biswas S, Deshpande PP, Navarro G, Dodwadkar NS, Torchilin VP (2013) Lipid modified triblock PAMAM-based nanocarriers for siRNA drug co-delivery. Biomaterials 34: 1289-1301.

6. Boswell CA, Eck PK, Regino CA, Bernardo M, Wong KJ, et al. (2008) Synthesis, characterization, and biological evaluation of integrin alphavbeta3-targeted PAMAM dendrimers. Mol Pharm 5: 527-539.

7. Cason CA, Fabre TA, Buhrlage A, Haik KL, Bullen HA (2012) Low-Level Detection of Poly(amidoamine) PAMAM Dendrimers Using Immunoimaging Scanning Probe Microscopy. Int J Anal Chem 2012: 341260.

8. Chanyshev B, Shainberg A, Isak A, Litinsky A, Chepurko Y, et al. (2012) Anti-ischemic effects of multivalent dendrimeric A(3) adenosine receptor agonists in cultured cardiomyocytes and in the isolated rat heart. Pharmacol Res 65: 338-346.

9. Chen X, Dou S, Liu G, Liu X, Wang Y, et al. (2008) Synthesis and in vitro characterization of a dendrimer-MORF conjugate for amplification pretargeting. Bioconjug Chem 19: 1518-1525.

10. Chen Y, Wang G, Kong D, Zhang Z, Yang K, et al. (2012) In vitro and in vivo double-enhanced suicide gene therapy mediated by generation 5 polyamidoamine dendrimers for PC-3 cell line. World J Surg Oncol 10: 3.

11. Choi SK, Thomas T, Li MH, Kotlyar A, Desai A, et al. (2010) Light-controlled release of caged doxorubicin from folate receptor-targeting PAMAM dendrimer nanoconjugate. Chem Commun (Camb) 46: 2632-2634.

12. Dai H, Navath RS, Balakrishnan B, Guru BR, Mishra MK, et al. (2010) Intrinsic targeting of inflammatory cells in the brain by polyamidoamine dendrimers upon subarachnoid administration. Nanomedicine (Lond) 5: 1317-1329.

13. Desai PN, Yuan Q, Yang H (2010) Synthesis and characterization of photocurable polyamidoamine dendrimer hydrogels as a versatile platform for tissue engineering and drug delivery. Biomacromolecules 11: 666-673.

14. Ellis GA, Hornung ML, Raines RT (2011) Potentiation of ribonuclease cytotoxicity by a poly(amidoamine) dendrimer. Bioorg Med Chem Lett 21: 2756-2758.

15. Filipowicz A, Wolowiec S (2012) Bioconjugates of PAMAM dendrimers with trans-retinal, pyridoxal, and pyridoxal phosphate. Int J Nanomedicine 7: 4819-4828.

16. Goldberg DS, Vijayalakshmi N, Swaan PW, Ghandehari H (2011) G3.5 PAMAM dendrimers enhance transepithelial transport of SN38 while minimizing gastrointestinal toxicity. J Control Release 150: 318-325.

17. Guo S, Huang Y, Wei T, Zhang W, Wang W, et al. (2011) Amphiphilic and biodegradable methoxy polyethylene glycol-block-(polycaprolactone-graft-poly(2-(dimethylamino)ethyl methacrylate)) as an effective gene carrier. Biomaterials 32: 879-889.

18. Herd HL, Malugin A, Ghandehari H (2011) Silica nanoconstruct cellular toleration threshold in vitro. J Control Release 153: 40-48.

19. Huang R, Ke W, Han L, Li J, Liu S, et al. (2011) Targeted delivery of chlorotoxin-modified DNA-loaded nanoparticles to glioma via intravenous administration. Biomaterials 32: 2399-2406.

20. Intra J, Salem AK (2010) Fabrication, characterization and in vitro evaluation of poly(D,L-lactide-co-glycolide) microparticles loaded with polyamidoamine-plasmid DNA dendriplexes for applications in nonviral gene delivery. J Pharm Sci 99: 368-384.

21. Kailasan A, Yuan Q, Yang H (2010) Synthesis and characterization of thermoresponsive polyamidoamine-polyethylene glycol-poly(D,L-lactide) core-shell nanoparticles. Acta Biomater 6: 1131-1139.

22. Kamaly N, Miller AD (2010) Paramagnetic liposome nanoparticles for cellular and tumour imaging. Int J Mol Sci 11: 1759-1776.

23. Kang SJ, Durairaj C, Kompella UB, O'Brien JM, Grossniklaus HE (2009) Subconjunctival nanoparticle carboplatin in the treatment of murine retinoblastoma. Arch Ophthalmol 127: 1043-1047.

24. Kannan S, Dai H, Navath RS, Balakrishnan B, Jyoti A, et al. (2012) Dendrimer-based postnatal therapy for neuroinflammation and cerebral palsy in a rabbit model. Sci Transl Med 4: 130ra146.

25. Kecskes A, Tosh DK, Wei Q, Gao ZG, Jacobson KA (2011) GPCR ligand dendrimer (GLiDe) conjugates: adenosine receptor interactions of a series of multivalent xanthine antagonists. Bioconjug Chem 22: 1115-1127.

26. Kim Y, Hechler B, Gao ZG, Gachet C, Jacobson KA (2009) PEGylated dendritic unimolecular micelles as versatile carriers for ligands of G protein-coupled receptors. Bioconjug Chem 20: 1888-1898.

27. Kim Y, Klutz AM, Jacobson KA (2008) Systematic investigation of polyamidoamine dendrimers surface-modified with poly(ethylene glycol) for drug delivery applications: synthesis, characterization, and evaluation of cytotoxicity. Bioconjug Chem 19: 1660-1672.

28. Kojima C, Toi Y, Harada A, Kono K (2007) Preparation of poly(ethylene glycol)-attached dendrimers encapsulating photosensitizers for application to photodynamic therapy. Bioconjug Chem 18: 663-670.

29. Kojima C, Turkbey B, Ogawa M, Bernardo M, Regino CA, et al. (2011) Dendrimer-based MRI contrast agents: the effects of PEGylation on relaxivity and pharmacokinetics. Nanomedicine (Lond) 7: 1001-1008.

30. Kolhatkar RB, Swaan P, Ghandehari H (2008) Potential oral delivery of 7-ethyl-10-hydroxy-camptothecin (SN-38) using poly(amidoamine) dendrimers. Pharm Res 25: 1723-1729.

31. Kukowska-Latallo JF, Candido KA, Cao Z, Nigavekar SS, Majoros IJ, et al. (2005) Nanoparticle targeting of anticancer drug improves therapeutic response in animal model of human epithelial cancer. Cancer Res 65: 5317-5324.

32. Kuo JH, Liou MJ, Chiu HC (2010) Evaluating the gene-expression profiles of HeLa cancer cells treated with activated and nonactivated poly(amidoamine) dendrimers, and their DNA complexes. Mol Pharm 7: 805-814.

33. Lai P, Lou P, Peng C, Pai C, Yen W, et al. (2007) Doxorubicin delivery by polyamidoamine dendrimer conjugation and photochemical internalization for cancer therapy. Journal of Controlled Release 122: 39-46.

34. Lai PS, Pai CL, Peng CL, Shieh MJ, Berg K, et al. (2008) Enhanced cytotoxicity of saporin by polyamidoamine dendrimer conjugation and photochemical internalization. J Biomed Mater Res A 87: 147-155.

35. Li MH, Choi SK, Thomas TP, Desai A, Lee KH, et al. (2012) Dendrimer-based multivalent methotrexates as dual acting nanoconjugates for cancer cell targeting. Eur J Med Chem 47: 560-572.

36. Liu D, Hu H, Zhang J, Zhao X, Tang X, et al. (2011) Drug pH-sensitive release in vitro and targeting ability of polyamidoamine dendrimer complexes for tumor cells. Chem Pharm Bull (Tokyo) 59: 63-71.

37. Liu J, Liu J, Chu L, Wang Y, Duan Y, et al. (2011) Novel peptide-dendrimer conjugates as drug carriers for targeting nonsmall cell lung cancer. Int J Nanomedicine 6: 59-69.

38. Liu X, Huang H, Wang J, Wang C, Wang M, et al. (2011) Dendrimers-delivered short hairpin RNA targeting hTERT inhibits oral cancer cell growth in vitro and in vivo. Biochem Pharmacol 82: 17-23.

39. Liu X, Liu C, Laurini E, Posocco P, Pricl S, et al. (2012) Efficient delivery of sticky siRNA and potent gene silencing in a prostate cancer model using a generation 5 triethanolamine-core PAMAM dendrimer. Mol Pharm 9: 470-481.

40. Liu XX, Rocchi P, Qu FQ, Zheng SQ, Liang ZC, et al. (2009) PAMAM dendrimers mediate siRNA delivery to target Hsp27 and produce potent antiproliferative effects on prostate cancer cells. ChemMedChem 4: 1302-1310.

41. Mandeville JS, Bourassa P, Thomas TJ, Tajmir-Riahi HA (2012) Biogenic and synthetic polyamines bind cationic dendrimers. PLoS One 7: e36087.

42. Markowicz M, Szymanski P, Ciszewski M, Klys A, Mikiciuk-Olasik E (2012) Evaluation of poly(amidoamine) dendrimers as potential carriers of iminodiacetic derivatives using solubility studies and 2D-NOESY NMR spectroscopy. J Biol Phys 38: 637-656.

43. Maruyama-Tabata H, Harada Y, Matsumura T, Satoh E, Cui F, et al. (2000) Effective suicide gene therapy in vivo by EBV-based plasmid vector coupled with polyamidoamine dendrimer. Gene Ther 7: 53-60.

44. McIntyre JO, Fingleton B, Wells KS, Piston DW, Lynch CC, et al. (2004) Development of a novel fluorogenic proteolytic beacon for in vivo detection and imaging of tumour-associated matrix metalloproteinase-7 activity. Biochem J 377: 617-628.

45. Menjoge AR, Navath RS, Asad A, Kannan S, Kim CJ, et al. (2010) Transport and biodistribution of dendrimers across human fetal membranes: implications for intravaginal administration of dendrimer-drug conjugates. Biomaterials 31: 5007-5021.

46. Menjoge AR, Rinderknecht AL, Navath RS, Faridnia M, Kim CJ, et al. (2011) Transfer of PAMAM dendrimers across human placenta: prospects of its use as drug carrier during pregnancy. J Control Release 150: 326-338.

47. Merkel OM, Mintzer MA, Librizzi D, Samsonova O, Dicke T, et al. (2010) Triazine dendrimers as nonviral vectors for in vitro and in vivo RNAi: the effects of peripheral groups and core structure on biological activity. Mol Pharm 7: 969-983.

48. Mo Y, Barnett ME, Takemoto D, Davidson H, Kompella UB (2007) Human serum albumin nanoparticles for efficient delivery of Cu, Zn superoxide dismutase gene. Mol Vis 13: 746-757.

49. Mullen DG, Fang M, Desai A, Baker JR, Orr BG, et al. (2010) A quantitative assessment of nanoparticle-ligand distributions: implications for targeted drug and imaging delivery in dendrimer conjugates. ACS Nano 4: 657-670.

50. Mullen DG, McNerny DQ, Desai A, Cheng XM, Dimaggio SC, et al. (2011) Design, synthesis, and biological functionality of a dendrimer-based modular drug delivery platform. Bioconjug Chem 22: 679-689.

51. Myc A, Kukowska-Latallo J, Cao P, Swanson B, Battista J, et al. (2010) Targeting the efficacy of a dendrimer-based nanotherapeutic in heterogeneous xenograft tumors in vivo. Anticancer Drugs 21: 186-192.

52. Navarro G, Tros de Ilarduya C (2009) Activated and non-activated PAMAM dendrimers for gene delivery in vitro and in vivo. Nanomedicine (Lond) 5: 287-297.

53. Navath RS, Kurtoglu YE, Wang B, Kannan S, Romero R, et al. (2008) Dendrimer-drug conjugates for tailored intracellular drug release based on glutathione levels. Bioconjug Chem 19: 2446-2455.

54. Navath RS, Menjoge AR, Wang B, Romero R, Kannan S, et al. (2010) Amino acid-functionalized dendrimers with heterobifunctional chemoselective peripheral groups for drug delivery applications. Biomacromolecules 11: 1544-1563.

55. Nie Y, Ji L, Ding H, Xie L, Li L, et al. (2012) Cholesterol derivatives based charged liposomes for doxorubicin delivery: preparation, in vitro and in vivo characterization. Theranostics 2: 1092-1103.

56. Nomani A, Haririan I, Rahimnia R, Fouladdel S, Gazori T, et al. (2010) Physicochemical and biological properties of self-assembled antisense/poly(amidoamine) dendrimer nanoparticles: the effect of dendrimer generation and charge ratio. Int J Nanomedicine 5: 359-369.

57. Pan B, Cui D, Sheng Y, Ozkan C, Gao F, et al. (2007) Dendrimer-modified magnetic nanoparticles enhance efficiency of gene delivery system. Cancer Res 67: 8156-8163.

58. Patil ML, Zhang M, Minko T (2011) Multifunctional triblock Nanocarrier (PAMAM-PEG-PLL) for the efficient intracellular siRNA delivery and gene silencing. ACS Nano 5: 1877-1887.

59. Patil ML, Zhang M, Taratula O, Garbuzenko OB, He H, et al. (2009) Internally cationic polyamidoamine PAMAM-OH dendrimers for siRNA delivery: effect of the degree of quaternization and cancer targeting. Biomacromolecules 10: 258-266.

60. Perez AP, Cosaka ML, Romero EL, Morilla MJ (2011) Uptake and intracellular traffic of siRNA dendriplexes in glioblastoma cells and macrophages. Int J Nanomedicine 6: 2715-2728.

61. Perez-Martinez FC, Carrion B, Lucio MI, Rubio N, Herrero MA, et al. (2012) Enhanced docetaxel-mediated cytotoxicity in human prostate cancer cells through knockdown of cofilin-1 by carbon nanohorn delivered siRNA. Biomaterials 33: 8152-8159.

62. Pisal DS, Yellepeddi VK, Kumar A, Kaushik RS, Hildreth MB, et al. (2008) Permeability of surface-modified polyamidoamine (PAMAM) dendrimers across Caco-2 cell monolayers. Int J Pharm 350: 113-121.

63. Ren Y, Zhou X, Mei M, Yuan XB, Han L, et al. (2010) MicroRNA-21 inhibitor sensitizes human glioblastoma cells U251 (PTEN-mutant) and LN229 (PTEN-wild type) to taxol. BMC Cancer 10: 27.

64. Sadekar S, Ray A, Janat-Amsbury M, Peterson CM, Ghandehari H (2011) Comparative biodistribution of PAMAM dendrimers and HPMA copolymers in ovarian-tumor-bearing mice. Biomacromolecules 12: 88-96.

65. Santra S, Kaittanis C, Perez JM (2010) Aliphatic hyperbranched polyester: a new building block in the construction of multifunctional nanoparticles and nanocomposites. Langmuir 26: 5364-5373.

66. Sarin H, Kanevsky AS, Wu H, Brimacombe KR, Fung SH, et al. (2008) Effective transvascular delivery of nanoparticles across the blood-brain tumor barrier into malignant glioma cells. J Transl Med 6: 80.

67. Schilrreff P, Mundina-Weilenmann C, Romero EL, Morilla MJ (2012) Selective cytotoxicity of PAMAM G5 core--PAMAM G2.5 shell tecto-dendrimers on melanoma cells. Int J Nanomedicine 7: 4121-4133.

68. Shao N, Su Y, Hu J, Zhang J, Zhang H, et al. (2011) Comparison of generation 3 polyamidoamine dendrimer and generation 4 polypropylenimine dendrimer on drug loading, complex structure, release behavior, and cytotoxicity. Int J Nanomedicine 6: 3361-3372.

69. Shi X, Majoros IJ, Baker JR, Jr. (2005) Capillary electrophoresis of poly(amidoamine) dendrimers: from simple derivatives to complex multifunctional medical nanodevices. Mol Pharm 2: 278-294.

70. Shi X, Wang SH, Lee I, Shen M, Baker JR, Jr. (2009) Comparison of the internalization of targeted dendrimers and dendrimer-entrapped gold nanoparticles into cancer cells. Biopolymers 91: 936-942.

71. Shukla S, Wu G, Chatterjee M, Yang W, Sekido M, et al. (2003) Synthesis and biological evaluation of folate receptor-targeted boronated PAMAM dendrimers as potential agents for neutron capture therapy. Bioconjug Chem 14: 158-167.

72. Smith PE, Brender JR, Durr UH, Xu J, Mullen DG, et al. (2010) Solid-state NMR reveals the hydrophobic-core location of poly(amidoamine) dendrimers in biomembranes. J Am Chem Soc 132: 8087-8097.

73. Sweet DM, Kolhatkar RB, Ray A, Swaan P, Ghandehari H (2009) Transepithelial transport of PEGylated anionic poly(amidoamine) dendrimers: implications for oral drug delivery. J Control Release 138: 78-85.

74. Tekade RK, Kumar PV, Jain NK (2009) Dendrimers in oncology: an expanding horizon. Chem Rev 109: 49-87.

75. Thiagarajan G, Ray A, Malugin A, Ghandehari H (2010) PAMAM-camptothecin conjugate inhibits proliferation and induces nuclear fragmentation in colorectal carcinoma cells. Pharm Res 27: 2307-2316.

76. Thomas TP, Choi SK, Li MH, Kotlyar A, Baker JR, Jr. (2010) Design of riboflavin-presenting PAMAM dendrimers as a new nanoplatform for cancer-targeted delivery. Bioorg Med Chem Lett 20: 5191-5194.

77. Thomas TP, Majoros I, Kotlyar A, Mullen D, Holl MM, et al. (2009) Cationic poly(amidoamine) dendrimer induces lysosomal apoptotic pathway at therapeutically relevant concentrations. Biomacromolecules 10: 3207-3214.

78. Tosh DK, Phan K, Deflorian F, Wei Q, Yoo LS, et al. (2012) Click modification in the N6 region of A3 adenosine receptor-selective carbocyclic nucleosides for dendrimeric tethering that preserves pharmacophore recognition. Bioconjug Chem 23: 232-247.

79. Tosh DK, Yoo LS, Chinn M, Hong K, Kilbey SM, 2nd, et al. (2010) Polyamidoamine (PAMAM) dendrimer conjugates of "clickable" agonists of the A3 adenosine receptor and coactivation of the P2Y14 receptor by a tethered nucleotide. Bioconjug Chem 21: 372-384.

80. Tse C, Zohdy MJ, Ye JY, O'Donnell M, Lesniak W, et al. (2011) Enhanced optical breakdown in KB cells labeled with folate-targeted silver-dendrimer composite nanodevices. Nanomedicine (Lond) 7: 97-106.

81. Vijayalakshmi N, Ray A, Malugin A, Ghandehari H (2010) Carboxyl-terminated PAMAM-SN38 conjugates: synthesis, characterization, and in vitro evaluation. Bioconjug Chem 21: 1804-1810.

82. Waite CL, Roth CM (2009) PAMAM-RGD conjugates enhance siRNA delivery through a multicellular spheroid model of malignant glioma. Bioconjug Chem 20: 1908-1916.

83. Waite CL, Roth CM (2011) Binding and transport of PAMAM-RGD in a tumor spheroid model: the effect of RGD targeting ligand density. Biotechnol Bioeng 108: 2999-3008.

84. Wan TC, Tosh DK, Du L, Gizewski ET, Jacobson KA, et al. (2011) Polyamidoamine (PAMAM) dendrimer conjugate specifically activates the A3 adenosine receptor to improve post-ischemic/reperfusion function in isolated mouse hearts. BMC Pharmacol 11: 11.

85. Wang P, Zhao XH, Wang ZY, Meng M, Li X, et al. (2010) Generation 4 polyamidoamine dendrimers is a novel candidate of nano-carrier for gene delivery agents in breast cancer treatment. Cancer Lett 298: 34-49.

86. Wangler C, Moldenhauer G, Eisenhut M, Haberkorn U, Mier W (2008) Antibody-dendrimer conjugates: the number, not the size of the dendrimers, determines the immunoreactivity. Bioconjug Chem 19: 813-820.

87. Ward BB, Dunham T, Majoros IJ, Baker JR, Jr. (2011) Targeted dendrimer chemotherapy in an animal model for head and neck squamous cell carcinoma. J Oral Maxillofac Surg 69: 2452-2459.

88. Wilbur DS, Pathare PM, Hamlin DK, Buhler KR, Vessella RL (1998) Biotin reagents for antibody pretargeting. 3. Synthesis, radioiodination, and evaluation of biotinylated starburst dendrimers. Bioconjug Chem 9: 813-825.

89. Wu X, Ding B, Gao J, Wang H, Fan W, et al. (2011) Second-generation aptamer-conjugated PSMA-targeted delivery system for prostate cancer therapy. Int J Nanomedicine 6: 1747-1756.

90. Xu Q, Wang CH, Pack DW (2010) Polymeric carriers for gene delivery: chitosan and poly(amidoamine) dendrimers. Curr Pharm Des 16: 2350-2368.

91. Yang W, Barth RF, Wu G, Kawabata S, Sferra TJ, et al. (2006) Molecular targeting and treatment of EGFRvIII-positive gliomas using boronated monoclonal antibody L8A4. Clin Cancer Res 12: 3792-3802.

92. Yin Z, Liu N, Ma M, Wang L, Hao Y, et al. (2012) A novel EGFR-targeted gene delivery system based on complexes self-assembled by EGF, DNA, and activated PAMAM dendrimers. Int J Nanomedicine 7: 4625-4635.

93. Yuan Q, Lee E, Yeudall WA, Yang H (2010) Dendrimer-triglycine-EGF nanoparticles for tumor imaging and targeted nucleic acid and drug delivery. Oral Oncol 46: 698-704.

94. Yuan Q, Yeudall WA, Yang H (2010) PEGylated polyamidoamine dendrimers with bis-aryl hydrazone linkages for enhanced gene delivery. Biomacromolecules 11: 1940-1947.

95. Zhang L, Zhu S, Qian L, Pei Y, Qiu Y, et al. (2011) RGD-modified PEG-PAMAM-DOX conjugates: in vitro and in vivo studies for glioma. Eur J Pharm Biopharm 79: 232-240.

96. Zhang M, Guo R, Wang Y, Cao X, Shen M, et al. (2011) Multifunctional dendrimer/combretastatin A4 inclusion complexes enable in vitro targeted cancer therapy. Int J Nanomedicine 6: 2337-2349.

97. Zhang Y, Thomas TP, Desai A, Zong H, Leroueil PR, et al. (2010) Targeted Dendrimeric Anticancer Prodrug: A Methotrexate-Folic Acid-Poly(amidoamine) Conjugate and a Novel, Rapid, "One Pot" Synthetic Approach. Bioconjug Chem.

98. Zhang Y, Thomas TP, Lee KH, Li M, Zong H, et al. (2011) Polyvalent saccharide-functionalized generation 3 poly(amidoamine) dendrimer-methotrexate conjugate as a potential anticancer agent. Bioorg Med Chem 19: 2557-2564.

99. Zhou J, Neff CP, Liu X, Zhang J, Li H, et al. (2011) Systemic administration of combinatorial dsiRNAs via nanoparticles efficiently suppresses HIV-1 infection in humanized mice. Mol Ther 19: 2228-2238.

100. Zhu S, Hong M, Zhang L, Tang G, Jiang Y, et al. (2010) PEGylated PAMAM dendrimer-doxorubicin conjugates: in vitro evaluation and in vivo tumor accumulation. Pharm Res 27: 161-174.
